# Potential Therapeutic Targets of Quercetin, a Plant Flavonol, and Its Role in the Therapy of Various Types of Cancer through the Modulation of Various Cell Signaling Pathways

**DOI:** 10.3390/molecules26051315

**Published:** 2021-03-01

**Authors:** Saleh A. Almatroodi, Mohammed A. Alsahli, Ahmad Almatroudi, Amit Kumar Verma, Abdulaziz Aloliqi, Khaled S. Allemailem, Amjad Ali Khan, Arshad Husain Rahmani

**Affiliations:** 1 Department of Medical Laboratories, College of Applied Medical Sciences, Qassim University, Buraydah 51542, Saudi Arabia; smtrody@qu.edu.sa (S.A.A.); shly@qu.edu.sa (M.A.A.); aamtrody@qu.edu.sa (A.A.); k.allemailem@qu.edu.sa (K.S.A.); 2 Department of Biotechnology, Jamia Millia Islamia, New Delhi 51542, India; amitvrm999@gmail.com; 3 Department of Medical Biotechnology, College of Applied Medical Sciences, Qassim University, Buraydah 51542, Saudi Arabia; aaalieky@qu.edu.sa; 4 Department of Basic Health Sciences, College of Applied Medical Sciences, Qassim University, Buraydah 51542, Saudi Arabia; akhan@qu.edu.sa

**Keywords:** quercetin, polyphenolic flavonoids, natural chemopreventers, cell signaling pathways, anticancer activity, bioavailability

## Abstract

Polyphenolic flavonoids are considered natural, non-toxic chemopreventers, which are most commonly derived from plants, fruits, and vegetables. Most of these polyphenolics exhibit remarkable antioxidant, anti-inflammatory, and anticancer properties. Quercetin (Qu) is a chief representative of these polyphenolic compounds, which exhibits excellent antioxidant and anticancer potential, and has attracted the attention of researchers working in the area of cancer biology. Qu can regulate numerous tumor-related activities, such as oxidative stress, angiogenesis, cell cycle, tumor necrosis factor, proliferation, apoptosis, and metastasis. The anticancer properties of Qu mainly occur through the modulation of vascular endothelial growth factor (VEGF), apoptosis, phosphatidyl inositol-3-kinase (P13K)/Akt (proteinase-kinase B)/mTOR (mammalian target of rapamycin), MAPK (mitogen activated protein kinase)/ERK1/2 (extracellular signal-regulated kinase 1/2), and Wnt/β-catenin signaling pathways. The anticancer potential of Qu is documented in numerous in vivo and in vitro studies, involving several animal models and cell lines. Remarkably, this phytochemical possesses toxic activities against cancerous cells only, with limited toxic effects on normal cells. In this review, we present extensive research investigations aimed to discuss the therapeutic potential of Qu in the management of different types of cancers. The anticancer potential of Qu is specifically discussed by focusing its ability to target specific molecular signaling, such as p53, epidermal growth factor receptor (EGFR), VEGF, signal transducer and activator of transcription (STAT), PI3K/Akt, and nuclear factor kappa B (NF-*κ*B) pathways. The anticancer potential of Qu has gained remarkable interest, but the exact mechanism of its action remains unclear. However, this natural compound has great pharmacological potential; it is now believed to be a complementary—or alternative—medicine for the prevention and treatment of different cancers.

## 1. Introduction

Cancer prevalence and associated mortality accounts for more than 18.1 million new cases worldwide and 9.6 million deaths, respectively [1]. While men most commonly suffer from colorectal, lung, prostate, liver, and stomach cancers, common cancer in women are cervical, thyroid, colorectal, and lung [2]. Several factors can lead to the development of cancer, which can range from endogenous factors, such as age or genetic predisposition, inherited mutations, hormones, and immune conditions or exogenous factors, or acquired factors, such as environment, lifestyle, nutrition, tobacco, diet, obesity, exposure to sun, radiation, chemicals and infectious organisms, or both [3,4,5]. Numerous indicators of cancer advancement and proliferation have been suggested by studies in the past to mark its growth, which includes metastasis, inhibition of cell death, increased invasion, maintenance of proliferation, replicative immortality, evasion of growth inhibitory factors, and angiogenesis [6,7]. Remarkable progress has been made in the last decade in understanding the hallmark capabilities of cancer, which have aided in early detection along with treatment modalities, and expanded the domain of cancer inhibition and prevention [8]. Traditionally, therapeutic modalities for cancer treatment have been available, which includes surgery, radiotherapy, chemotherapy, and immunotherapy used either individually or in combination [9,10]. However, these methods, besides being rigorous in nature, often evidence obstruction in efficiency over the course of treatment, primarily due to cancer drug inducted resistance, and cancer cells adapting to chemotherapeutic agents in the later stages of drug administration [11].

Improvement in healthcare facilities, diagnostic tools, advancement in treatment approaches, awareness, and accessibility of people towards cancer, especially in the past decade, have shown improvement in its prevention and cure over the years [12,13,14], yet its prevalence and mortality has grown steadily. The increasing prevalence of cancer has caused high mortality, and, besides this, the lower efficacies of existing treatment therapies need a better preventive strategy in its management [15,16]. Therefore, the growth of adjuvant or alternative drugs have become imperative over the years in the field of cancer prevention with the technological advancement and knowledge expansion. Presently, along with traditional cancer prevention techniques, there has been an upsurge in the use of complementary and alternative medicine (CAM) and natural health products [17,18,19]. As part of cancer chemoprevention, long-term intervention with natural or synthetic molecules are being experimented significantly, representing a potential area of natural dietary factors and showing remarkable evidence to prevent or reverse oncogenesis process, or can efficiently influence the cancer risk among human beings [17,20]. Scientific evidences and observational studies from the past indicate that dietary factors, lifestyle, and food consumption patterns, especially the ones inclusive of a vegetable and fruit-rich diet, are associated with substantially altering the natural history of carcinogenesis and inhibiting certain types of cancers, such as colorectal, stomach, lung, and esophageal cancers [21,22,23,24]. 

**Figure 1:**
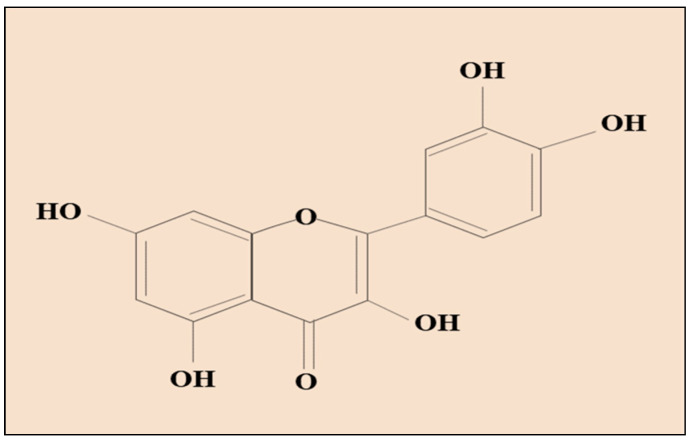
Chemical structure of quercetin [43].

Qu is a plant flavanol, which is commonly found in fruits, vegetables, and beverages, such as grapes [25], oranges [26], strawberries [27], onions [28], tomatoes [29], peppers [30], lettuce [31], radish leaves [32], papaya [33], almonds and pistachios [34], and nuts [35], with a chemical structure shown in Figure 1. Qu has been named as 3,3,4,5,7-pentahydroxyflavone by the International Union of Pure and Applied Chemistry (IUPAC) [36,37]. Another name for this agent, based on its typical characteristic is 3,3,4,5,7-pentahydroxy-2-phenylchromen-4-one, where presence of five hydroxyl groups at the 3-,5-,7-, and 4-positions can be observed [38].

An extensive range of pharmacological activities, such as anticancer, anti-inflammatory, antioxidant, and antimicrobial activities, have been attributed to Qu [39,40]. It has also been observed to act as anti-ulcer, anti-allergy, antitumor, anti-viral, and antidiabetic agents, presenting anti-hypertension, anti-infection, gastro-protection, and immune-modulation features, based on the extensive research conducted in the last decade under the umbrella of sustainable chemoprevention practices [41,42]. 

Several cancer-related molecules and conditions, such as antiapoptotic and proapoptotic proteins, and matrix metalloproteinase (MMPs), have been observed to be modulated by such biochemical–pharmacologic properties of Qu [42]. A significant impact has been observed due to its binding abilities, where it can potentially bind with G-protein-coupled receptors and activate a calcium-dependent pathway and a G-protein leading to death of the tumor cell inhibiting its progression [44,45,46]. The study of Qu is receiving increasing importance as a potential chemopreventer, due to its potential engagement in repressing many cancer-related/tumor-related processes, such as oxidative stress, apoptosis, and metastasis. Qu is also gaining importance due to enhanced apoptotic effects on tumor cell lines [47]. 

Studies from the past also claim that Qu plays an effective role in several other events of cancer initiation and progression, such as epithelial to mesenchymal transition, cancer-associated inflammation, extracellular matrix remodeling, oxidative stress, and cell signaling pathway [48,49,50]. Qu was evidenced to act against chemotherapeutic resistance in several cancers; thus, inhibiting its progression and/or even reversing it sometimes [44,45]. 

Because of extensive pharmacological and effective cancer inhibition properties and several health-promoting benefits of Qu, the current review article presents a cumulative compendium of extensive research investigating the potential therapeutic role of Qu in treatment of various types of cancers. Since it is asserted to carry antioxidant, antitumor, and anti-inflammatory properties, and has the ability to inhibit the proliferation of cancer cells, it has been extensively studied as a natural chemopreventive agent in several cancer models, and Qu modulates various cell signaling molecules.

## 2. Major Mechanism of Qu in Management of Cancer 

Qu plays a major role in the inhibition of cancer development and progression through modulation of various cell signaling pathways. The role of cell signaling pathways in the cancer management are discussed as follows. 

### 2.1. Inflammation

Inflammation is a biologically complex protective reaction of the body, arising due to harmful stimuli and damaged cells. Several disorders or conditions are characterized by inflammation, such as asthma, celiac disease, transplant rejection, allergy, hepatitis, preperfusion injury, glomerulonephritis, autoimmune diseases, inflammatory bowel disease, etc., along with cancer [51]. Therefore, inflammation is basically a self-protecting, biological reaction of the body in case of trouble, which, in the process, removes injured cell adverse stimuli, and begins the process of healing [52,53]. Chronic inflammation areas and damaged tissue sites have higher concentrations of immune sensitive cells (e.g., neutrophils, monocytes, and eosinophils), forming a microenvironment that may promote tumor invasion, metastasis, and angiogenesis [54]. Immunosuppression induced by chronic inflammation may contribute to cancer development [55]. Chemopreventers, such as Qu, may induce antioxidant activity; and can exert anti-inflammation and anti-proliferation actions. They may act as modulators of signal-transduction cascades, which are involved in approximately every biological process, such as an oxidative balance state, cell differentiation, apoptosis, cell proliferation, inflammation, and cell migration [56]. Qu, being a dietary antioxidant, also possesses anti-inflammatory properties and an inverse risk effect on several aero digestive cancers, by mitigating inflammation conditions [57,58]. 

**Figure 2:**
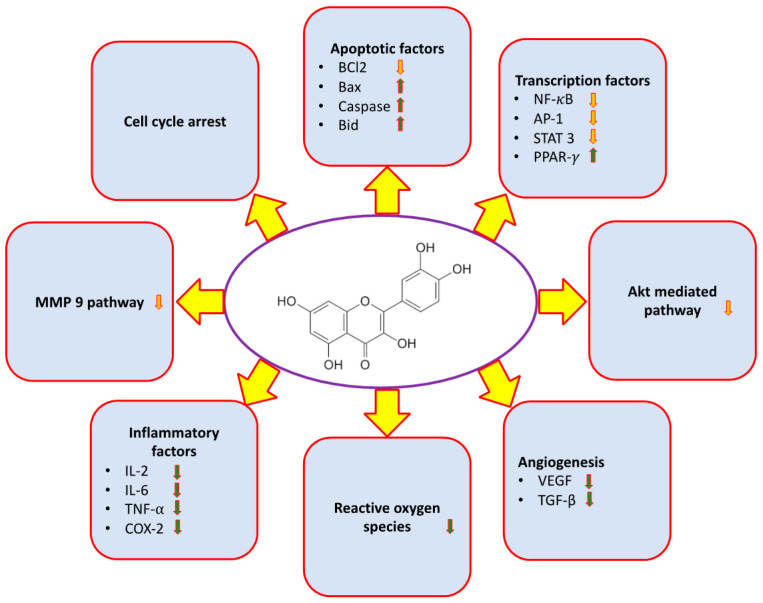
Quercetin shows role in the inhibition of cancer through the modulation of cell signaling molecules.

The ability of Qu to deal with inflammation is one of its critical and considerable features in cancer prevention (Table 1). The agent has been observed to inhibit the inflammatory enzymes of cyclooxygenase (COX) and lipoxygenase, thereby reducing inflammatory mediators [52,53]. Li et al. asserts—on the abilities of Qu—the discouragement of cancer development by hindering the development of inflammation-producing enzymes and reducing the production of pro-inflammatory mediators [59]. Qu has also been observed to intervene in nuclear factor kappa B (NF-*κ*B) pathway [60]. The NF-*κ*B family of transcription factors regulates several gene expressions involved in carcinogenesis, inflammation, and cytoprotection [61]. Thus, it plays a crucial role in innate immunity and inflammation; it is gradually considered as an important factor in several stages of cancer development [62]. NF-*κ*B along with activator protein 1 (AP-1) is involved in regulating expression of several genes that are associate with adaptation, differentiation, many cellular growth processes, cancer development, etc. [63]. Qu, having chemopreventive effects, is observed to suppress NF-*κ*B and AP1, which in turn impact various cancer-related processes [64]. The role of Qu in the inflammatory processes has been explained through the suppression of COX-2, NF-*κ*B, and AP-1 pathway (Figure 2).

The study by Youn et al. shows the role of Qu in NF-*κ*B inhibition leading to death receptors and cell cycle inhibitors, retarding growth of H460 lung cancer cells [65]. The Qu supplementation also impacts the condition of oxidative stress and inflammation in sarcoidosis, which have been assessed by several researchers. Boots et al., in their study, explained how Qu dosage on non-smoking, untreated sarcoidosis patients, increased total plasma antioxidant capacity and improved the antioxidant defense [66]. 

Another investigation affirmed that increased levels of C-reactive protein was associated with several cardiovascular and health problems, the risk of which can be significantly reduced by consumption of certain types of foods that are rich in anti-inflammation elements, such as phytochemicals and flavonoids, including Qu. In experiments conducted on the human hepatocyte-derived cell line, these elements were shown to reduce the level of inflammation moderators, NO synthase and COX-2 [67]. Inhibitory effects of Qu associated with prostate cancer have been demonstrated through several clinical studies, where it was shown to reduce inflammation and associated risk of prostate cancer [68,69]. In vitro experiments conducted on rats have also proved that administering specific Qu doses reduces acute and chronic inflammatory conditions [70,71]. 

Due to a wide range of anti-inflammatory properties, quercetin is widely studied as a chemoprevention agent in several cancer models.

### 2.2. Reactive Oxygen Species (ROS)

Persistence of reactive oxygen species (ROS) plays a vital role in the development and progression of various diseases, including cancer. Qu plays a role in the inhibition of pathogenesis through scavenging of ROS (Figure 2).

Excessive ROS is associated with high susceptibility of cancer cells to Qu, which in turn may reduce intracellular glutathione and boost intracellular ROS, to an extent, which can lead to cell death. Appropriate levels of ROS often modulate several physiologic responses, since it is important for signaling network regulating cell functions. However, the excessive intracellular levels of ROS encourage pathological conditions and the progression of diseased conditions [72]. One of the vital source of endogenous ROS is the mitochondrial respiratory chain, along with other oxidative enzymes, such as plasma membrane oxidases [73]. Qu plays a role in the inhibition of the pathogenesis of diseases through its ability as an antioxidant and scavenger of ROS, which offers this compound as a therapeutic module in disease management [74]. 

Ward et al. witnessed the impact of Qu in balancing ROS production in prostate cancer cells through its pronounced impact on mitochondrial integrity, and its antioxidant as well as pro-oxidant properties [75] (Table 1). Its chemical properties and structural composition creates an ideal arrangement for free radical-scavenging activity, exerting antioxidant effects and effectively scavenging ROS; thus, decreasing the ROS level, discouraging lipid peroxidation, and proving beneficial for cancer prohibition [42,76]. The role of Qu in balancing iron metabolism is also of significance, wherein Qu reduces high valent iron in order to inhibit lipid oxidation and ROS production and modulation of various molecules [77,78]. The role of Qu in the metabolism of ROS in cancer cells has been explained.

### 2.3. Angiogenesis

In general, angiogenesis is associated with repair of injured body cells and reproductive development through formation of new capillaries, which is facilitated by endostatin, growth factors, adhesion molecules, etc. [79]. However, in a dysregulated state, it may also be closely associated with neoplastic diseases and significantly affect the growth of metastasis of tumors. It is, in fact, considered as the initial and crucial step in the growth and metastasis of cancer, and other malignant tumors, since it enables the growing tumor to receive oxygen and nutrients [7,79]. Qu has proven anticancer activity through the inhibition of the angiogenesis process (Figure 2). It has been reported that Qu utilizes its anti-angiogenesis effects in several cancer types [80]. 

The role of Qu in cancer prevention has been explained through in vivo and in vitro studies. The nanoformulation of Qu has proven its role in the suppression of ovarian cancer through the inhibition of angiogenesis [81]. Moreover, the role of Qu in cancer prevention has been documented through modulating cell signaling pathways. Qu has been observed to affect several key molecules, such as vascular endothelial growth factor (VEGF), which plays a significant role in survival of endothelial cells, and can cause tumor angiogenesis [82,83].

Some derivatives of Qu, such as the permethylated form of Qu, show contradictory effects on angiogenesis and on tumor stimulated human primary retinal endothelial cells. It was observed to be a potential inhibitor of angiogenesis, both in vitro and ex vivo [84]. 

In several cancers models, such as prostate and breast cancer, Qu has been associated in mediating several angiogenesis pathways, including the Vascular Endothelial Growth Factor Receptor-2 mediated pathway of angiogenesis, repressing the downstream-regulatory target AkT expression; thus, restraining and restricting growth of the tumor [80,85].Lian Wang et al., through a mice model study, stated that Qu, as an angiogenesis inhibitor, reduces neovascularization growth, which is mediated via the inhibition of cyclooxygenase-2 (COX-2) [86]. It also decreases the expression of proangiogenic mediators, including VEGF-A, and Hypoxia-inducible factor 1-alpha; thus, preventing metastasis of cancer cells [87,88].

### 2.4. Apoptosis

Apoptosis is a process by which unhealthy and unrequired cells undergo programmed cell death to be removed from the body. It is, basically, the program of cell suicide, which removes virus infected cells and cancer cells, and retains balance in the body. It has been shown that Qu can influence the pathway of apoptosis and induce death of tumor cells. The anticancer role of Qu has been explained through the modulation of the apoptotic pathway; it stimulates the proapoptotic genes and inhibits the antiapoptotic genes (Figure 2). Qu causes cell death through intrinsic and extrinsic pathway of apoptosis [89]. 

While tumor cells elude apoptosis and progress, several phytochemicals, including Qu, have been observed to trigger apoptosis of tumor cells. Extrinsic pathway apoptosis has gained importance in anticancer study, since it is primarily associated with death of tumor cells and saving normal cells [90]. At the same time, Qu is also associated with stimulating intrinsic pathways and increases the release of cytochrome c from the mitochondria to the cytoplasm. Both processes are responsible for the death of tumor cells and inhibit chances of cancer progression [91]. Treatment with sufficient dosages of Qu has been demonstrated to enhance the proapoptotic proteins expression, reduce antiapoptotic proteins expression, and has been related to ovarian cancer cell apoptosis [81]. Similarly, Qu has been observed to induce apoptosis of gastric cancer stem cell [92], and have been found to be associated with tumor necrosis factor-related apoptosis-inducing ligand (TRAIL) to stimulate apoptosis in colon cancer, non-small cell lung cancer (NSCLC), glioma, and several other cancerous cells. It can collaborate with TRAIL to stimulate apoptosis in colon cancer, NSCLC, glioma, and several other cancerous cells through two approaches, by increasing the DR5 expression, and by preventing the expression of surviving [93,94]. Qu plays a role in apoptosis and manages the cancer.

### 2.5. Cell Cycle

A cell cycle is a repeating series of events, which involves copying of contents of cells and following division of cells. Cells are continuously subject to DNA mutation that is harmful for cells, but hardly results in cell production, which can avoid the normal restrictions, and flourish as pathologic tumors [95]. The development and progression of cancer is often associated with disruption or dysregulation of normal cell-cycle progression. Cells react to damage in DNA by stopping cell cycle progress and/or by enduring apoptosis [95].

Several flavonoids and natural chemopreventers, including Qu, have been observed to precisely regulate numerous proteins, which are involved in cellular homeostasis and cell cycle, and whose deregulation may play a role in carcinogenesis [96,97]. The ability of Qu to induce cell cycle arrest has been observed in a study conducted by Yuan et al. and Yoshida et al.; it shows that Qu therapy can lead to G0–G1 cell cycle arrest in leukemia [98,99], or S-phase arrest during cell cycle progression in tested cancer cells, such as what was observed in colorectal carcinoma [100,101] or G2-M phases of the cell cycle in esophageal adenocarcinoma, breast cancer, and leukemia cell lines [102,103,104].

He et al. carried out an investigation and showed that Qu inhibits proliferation of MM.1R and ARP-1 myeloma cell lines via causing apoptosis and G2-M phase cell cycle arrest [105]. An in vitro study conducted by Ma et al. has observed that the administration of 75 μM of Qu causes apoptosis and cell cycle arrest in stem cells of human colorectal cancerous cell lines, and enhances anticancer effects of doxorubicin [106]. Similarly, Long et al. performed a study on Qu and its impact on cisplatin resistance and sensitive ovarian cancer. The results showed that lipo-Qu caused apoptosis and cell cycle arrest and repressed proliferation of these cancer cells [87]. In another study conducted by Chang et al., it was revealed that Qu induces apoptosis, cell cycle arrest, and autophagy [107]. In an investigation conducted by Nguyen et al., based on triple negative breast cancer cells, it has been reported that Qu significantly induces apoptosis and causes cell cycle arrest. Qu impacts the FOXO-3a signaling pathway, which is involved in cell cycle arrest and apoptosis by regulating gene transcription involved in causing cell cycle arrest and apoptosis [108]. In a study conducted by Jeong et al., based on the administration of different doses of Qu, it has been reported that even low doses of Qu induces antiproliferative effects and prevents cell cycle progression [109].

### 2.6. Tumor Suppressor Gene

Qu plays a role in cancer prevention through the activation of the tumor suppressor gene. It has been considered as a proapoptotic flavonoid with specific activities on tumor cell lines [47]. Several clinical trials verified the safety of intravenous administration of Qu and observed its antitumor activities [110]. 

The effects of Qu on p53 or TP53 have been observed to be profound. Since Qu is a DNA intercalator, it may cause DNA damage, leading to p53 upregulation. This causes, in the downregulation of antiapoptotic protein, BCL-2, and cleavage of MCL-1 suppressing tumor growth. In vitro researches have confirmed that Qu efficiently prevents the growth of several prostate cancerous cell lines. When used in vivo, Qu prevents growth of xenograft prostate cancer [111]. It has also been used as a combination drug along with corticosteroids, such as dexamethasone, which has been observed to further enhance apoptosis and inhibit tumor growth [105].

### 2.7. Phosphatidylinositide-3-Kinase (PI3K)/Akt Pathways

Phosphatidylinositide-3-kinase (PI3K) is an important signal-transducing enzyme, which regulates cell differentiation, survival, angiogenesis, proliferation, and apoptosis [112,113]. The anticancer activity of Qu has been found through the inhibition of phosphatidylinositide-3-kinase (PI3K)/Akt pathways (Figure 2).

It is vital for Akt activation, which has an important role in pathological and physiological signaling processes. In a study by Maurya et al., Qu reduced Akt and PDK1 phosphorylation while upregulated the tumor suppressor phosphatase and tensin homolog (PTEN) level. This is constant with reduced levels of cell survival factors in Dalton’s lymphoma ascites (DLA) cells in vitro. Qu’s role in Dalton’s lymphoma ascites has been proven through the inhibition of the PI3K/Akt signaling pathway [114].

### 2.8. Signal Transducer and Activator of Transcription 3 (STAT3)

STAT3 is a part of the STAT family of transcription-factors and plays an important role in cancer-related inflammation. STAT3 is often deregulated in several kinds of cancer, and functions as an oncogene in tumorigenesis [115]. STAT3 activation causes expression of downstream genes, which regulate main cell responses, such as BCL2, cyclin-D1, and MMP-2 [116]. STAT3 plays an important role in tumorigenesis and in progression of cancer, which allows STAT3 to arise as a promising molecule target in the treatment of cancer. The anticancer activity of Qu has been found through the inhibition of the signal transducer and activator of transcription 3 (STAT3) (Figure 2).Qu has been reported to be capable of reducing glioblastoma cell proliferation and migration via the inhibition of STAT3 activation [115,117]. Further, it inhibits the proliferation of human gastric cancer cell line, which is related to downregulation of mRNA and proteins expression by JAK–STAT pathway [118]. Moreover, Qu’s role in cancer is reported through inhibitory activity against the STAT3 mechanism in diffuse big B-cell lymphoma cells [119]. Moreover, cardioprotective effects of Qu can be attained through decreasing STAT3 and Src kinase activities [120]. Qu was also found to prevent hepatocyte growth factor induced c-Met phosphorylation among human-medulloblastoma cell lines [121] and considerably prevented hepatocyte growth factor induced invasion and migration in cell line [121] and in human liver cancer cell line HepG2 [122].

Qu stimulates apoptosis through depolarization of mitochondria, causing imbalance in ratio of B cell lymphoma-2/Bcl-2 antagonist-X and through downregulating the interleukin-6/STAT3 signaling pathway. Furthermore, Qu can prevent action of NF-*κ*B in initial hours, which may trigger downregulation of interleukin-6 titer and expression of interleukin-6 can inhibit expression of p-STAT3. The downregulation of NF-*κ*B and STAT3 subsequently results in Bcl-2 downregulation, as both are upstream Bcl-2 effectors. Modifications in Bcl-2 reactions in the A549 cell line may cause an imbalance in the ratio of Bcl-2/Bax, which can ultimately lead to mitochondria-mediated apoptosis [123,124]. Seo et al. reported that, in breast cancer, Qu upregulates the cleaved caspase 8 and caspase 3 levels and suppresses p-STAT3 expressions, which results in decreasing STAT3 dependent luciferase-reporter activity of the gene [125].

### 2.9. Epidermal Growth Factor Receptor (EGFR)

EGFR is a part of a family of Erb-β (erythroblastic leukemia viral oncogene) of the tyrosine kinase receptor, which transmits growth-inducing signals to the cells, which have been controlled through the EGFR-ligand, such as EGF and *Transforming growth factor alpha* (*TGF-α*) [126]. EGFR signaling pathway activation stimulates downstream signaling cascades, involved in the antiapoptotic pathway (phosphatidylinositol 3-kinase PI3K/AKt) and in proliferation of the cell (Ras/mitogen-activated protein kinase (MAPK) [127]. EFG controls metastasis of cancer by controlling epithelial-mesenchymal transition (EMT) [128]. EMT transdifferentiation mechanism involves the transformation of adherent epithelial cells into single migratory cells, which causes change in the phenotype of the cells into more loose mesenchymal cells, encouraging metastatic dissemination and local invasion of tumor cells [129].

The role of Qu in prostate cancer is reported through the regulation of EFGR signaling and other molecules (e.g., cell adhesion, such as vimentin, N-cadherin, and E-cadherin). Qu effectively prevented carcinogenesis in dorsolateral (as well as in ventral) prostate. Supplementation of Qu considerably reduced the expression of proliferating cell nuclear antigen in both lobes of chemically induced cancerous mice [130]. In the in vitro model, Qu inhibits EGF-induced EMT and suppresses the transcriptional repressor slug, twist, and snail in prostrate cancerous cell line PC-3. Hence, Qu inhibits metastasis of cancer by aiming EMT [131]. Arunkaran J (2017), in his study, synthesized gold nanoparticle conjugated-Qu, which demonstrated efficient cytotoxicity and apoptosis induction in estrogen-independent and dependent breast cancerous cell lines [132]. Synthesized gold nanoparticles prevented the EGFR phosphorylation and downstream molecules of PI3K-Akt pathway in breast cancerous cell lines [133]. In breast cancer, AuNps-QU-5 prevents epithelial-mesenchymal transitions, invasiveness, and angiogenesis. Hence, several studies demonstrated that Qu is efficient in preventing cancers, such as breast and prostate cancer [132,133,134]. Bhat (2014) et al. observed that EGF-stimulated protein expression of EFGR, Akt, phospho-Akt, and it was considerably reduced by Qu [135]. Akt is a vital factor of cell survival, which controls the cell cycle progression [136]. Akt inhibits cells from enduring apoptosis by preventing pro apoptotic proteins, BAD and caspase-9, and Forkhead-transcription factor nuclear translocation [137]. Akt overexpression has been found in several cancers comprising prostate cancer as it favors cancerous cell angiogenesis, invasion, and proliferation [138]. Qu prevented EGF induced PI3-K and PDk-1 phosphorylation and, thus, downregulated protein levels of Akt [130].

## 3. Role of Qu in Prevention and Inhibition of Various Types of Cancer

**Figure 3:**
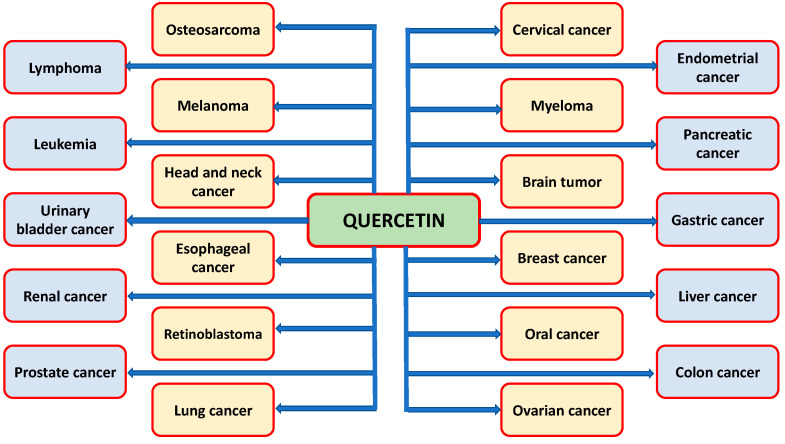
Quercetin has a proven anticancer role in multiple cancers, based on in vivo and in vitro studies.

**Figure 4:**
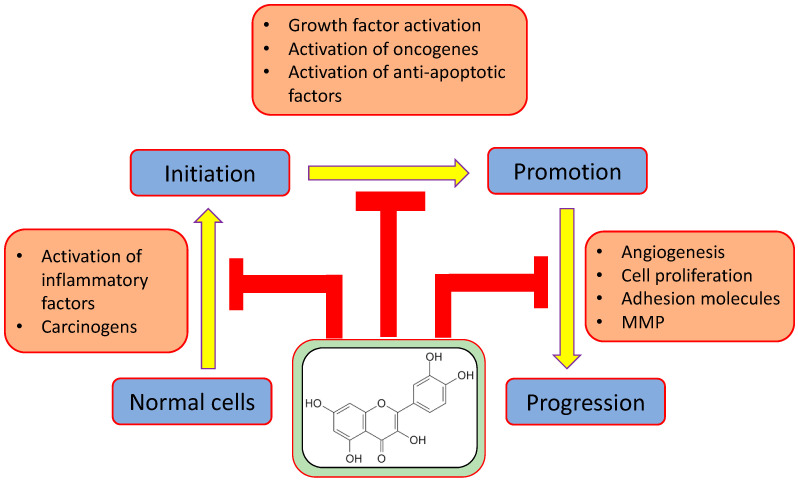
Anticancer activity of quercetin confirmed through the inhibition of carcinogenesis steps, including initiation, promotion, and progression.

The studies based on in vivo and in vitro has proven its role in cancer management through modulating various cell signaling pathways (Figure 3). Cancer is a multifactorial process, which included initiation, promotion, and progression. The activation of inflammatory factors and carcinogens convert normal cells to the initiation phase, which are further subject to the promotion phase. This transition is activated by growth factors and oncogenes, and antiapoptotic factors. The promotion phase is converted to the progression phase by angiogenesis, cell proliferation, and adhesion molecules. Qu’s role in cancer prevention has been confirmed through the inhibition of carcinogenesis steps, such as initiation, promotion, and progression (Figure 4). The role of quercetin has been proven in various types of cancer through modulating cell signalling molecules (Table 2).

### 3.1. Cervical Cancer

One of the most common gynecological cancers is cervical cancer, which accounts as a common cause of female deaths globally [171]. It is well established that the main etiological agent and initial stage of cervical cancer is influenced with persistent Hunan Papilloma virus (HPV) infection exposure [172]. Qu plays a vital role in inhibition and prevention of cancer through modulating various biological activities (Table 2). In a study conducted by Ali et al., it was concluded that Qu induces cell death in cervical cancer. Results show that Qu displays its anticancer effect by reducing O-GlcNAcylation, a key regulator of progression of cancer [139]. The study conducted by Kedhari et al. showed that Qu alters the WNT, PI3K, and MAPK pathways by regulating the expressions of various proteins, which leads to the inhibition of cell cycle arrest, cell proliferation, apoptosis, and DNA damage in cervical cancerous cells [140]. Administration of nanoparticles of Qu in cancerous cervical cells was done in a study conducted by Luo et al. The effect of Qu nanoparticles on cervical cancer progression was found to induce apoptosis, autophagy, and anti-proliferation [173]. In another study conducted by Clemente-Soto et al., it was also observed that Qu arrests the cell cycle in the G2-phase and apoptosis with p53 activation in an E6-expression independent manner [174]. Lin et al. showed that Qu decreases UBE2S expression, which is greatly expressed in malignant cancers, and contributes to cell motility via EMT signaling [175].

### 3.2. Breast Cancer

Breast cancer is one of the most prevalent cancers among females in the world, with a significantly high mortality rate. In spite of recent progress in early detection and therapeutic strategies, the prevalence and mortality rate has increased [176]. Recent studies have reported that Qu inhibits breast cancer by inhibiting signal transduction, inducing cancer cell apoptosis, and suppressing proliferation, invasion, and metastases of tumor cells [177,178]. Qu was also stated to repress the motility of breast and melanoma cancerous cells [85,179].

Chien et al. reported that Qu causes the death of human breast cancer cells via mitochondrial- and caspase-3-dependent pathways [56]. In breast cancers, Qu is also observed to target the VEGFR-2 mediated angiogenesis pathway, suppress the expression of the downstream regulatory factor AkT, and inhibit tumor growth [80,85]. 

Qu leads to apoptosis by cytochrome c from mitochondria, and, on the other hand, leads to generation of cancer stem cells (CSCs), which may reduce cancer recurrence [180].

### 3.3. Ovarian Cancer

Among all of the reproductive cancers, ovarian cancer is one of the most fatal among women. It is considered to be the fifth leading cause of cancer deaths worldwide among women [181], accounting for about 4% of female deaths globally [81]. Early diagnosis of ovarian cancer is difficult—despite widespread awareness among women—due to its silent and unclear symptoms and its late diagnosis at advanced stages, which makes it more life-threatening [182]. 

Several risk factors have been associated with ovarian cancer, including increasing age, null parity, early menarche, late menopause, family history, etc. [81]. Moreover, diet plays a significant role in increasing or decreasing risks of some malignancy [183], and evidence indicates that ovarian cancer has an inverse association with higher intakes of fruits and vegetables and vice versa [184]. Differences in molecular structure and genetic alterations with progression, diverse response to existing therapies, resistance of cancer cells to common cancer therapies in ovarian cancer, creates difficulty in designing specific therapeutic interventions and platforms [185]. Provisions of alternative medication calls for new therapeutic approaches in cancer prevention. Various phytochemicals have significant anticancer characteristics, which can be used against various cancer types [41]. Evidence from past research, in vitro and in vivo studies performed to assess cytotoxic effects of Qu on ovarian cancer, reveal that Qu, a plant flavonol, is able to inhibit various types of cancers, including ovarian cancer. 

Qu also possesses the ability to cross the cell membrane and intervene with several intracellular signaling pathways involved in chemoprevention. Another study investigated Qu’s impact on apoptosis in mice with ovarian cancer. The findings revealed that Qu could interfere with intracellular signaling pathway induced apoptosis through mitochondrial intrinsic and caspase dependent pathways. Moreover, Qu induced endoplasmic reticulum stress in ovarian tumors, resulting in mitochondrial-mediated apoptosis. Additionally, Qu was able to cause autophagy, which plays a protective role in ovarian cancer cells [141]. The ovulation theory of ovarian cancer indicates recurrent ovulation with repeated breakdown and repair of ovarian epithelium to be the cause of tumorigenesis [186]. The process is followed by severe inflammatory reaction during the recurrent follicle rupture and epithelium detected around the place of ovulation, and has more connections with inflammatory facilitators, such as oxidative stress, prostaglandins, and cytokines, which tend to have constant inflammation [187]. Several clinical investigations have proved Qu to be anti-inflammatory and can significantly reduce the levels of inflammation moderators [67,70]. Mamani et al. suggested that Qu inhibits tumor growth and angiogenesis and induces apoptosis [188]. Mamani et al. revealed that an 80 mg (or equivalent) dose of Qu delayed acute and chronic inflammatory conditions [71].

Another in vivo mice study (conducted by Gao et al., 2012) revealed intravenous administration of Quercetin into biodegradable monomethoxy poly(ethylene glycol)-poly(ε-caprolactone (Qu/MPEG-PCL) micelles, water soluble forms of Qu were able to significantly decrease tumor volume by 66.14% through inhibiting angiogenesis and cancer cell apoptosis in mice [81]. Similarly, Scambia et al. reported that Qu, alone and in combination with cis-diamminedichloroplatinum II (CDDP), as another chemotherapeutic agent on advanced stages of ovarian cancer, showed growth inhibition and high potential to improve anti-proliferative activity of CDDP in all cases [189]. Another in vitro and in vivo combination study evaluated the Qu effect in combination with irradiation on ovarian cancer. Qu’s action led to Endoplasmic reticulum (ER stress), increased expression of Bax, p21, and p53, reduced Bcl-2 expression, continued repair of DNA, and caused radio sensitization in ovarian cancerous cells [142]. In another study, according to the The half maximal inhibitory concentration (IC50) values, Qu concentration, required for 50% cell kill, showed that administration of Qu before treatment with cisplatin or oxaliplatin produced a synergistic inhibitory effect on human ovarian cancer cell growth, and could reduce drug-resistance [190]. Numerous researches showed that high doses of Qu are also able to cause apoptosis through death domain pathways in various cancerous cells [191]. Qu also influences a large range of molecules involved in cell cycle. Gupta et al. examined that Qu was capable of precisely binding tubulin and stimulating depolymerization of cellular microtubule, which cause cell cycle arrest [192]. Studies also suggest Qu could be a good candidate for adjuvant therapy in ovarian cancer treatment. Moreover, Qu increased antitumor effects of cisplatin through a xenograft mouse model, where cisplatin-treated mice, in combination with pre-treatment of Qu, reduced Bcl-2, and high apoptosis was detected in comparison to other groups [193]. Therefore, several studies suggest the existence of positive association between the dose-dependent inhibitory effects of Qu on human ovarian cancer cell proliferation [194]. 

### 3.4. Endometrial Cancer 

Endometrial cancer is the most common female gynecological cancer with increasing prevalence worldwide. Several risk factors, including sedentary life style, lack of physical mobility, obesity, age, alterations in endogenous hormone metabolism, etc., can be associated with it. Epidemiologic studies have demonstrated enhanced risk of endometrial cancer in pre- and post-menopausal women who have high plasma testosterone and androstenedione and in postmenopausal women who have high levels of estrone and estradiol [195,196]. In an investigation, Qu shows significant inhibition effects on malignant cell growth, including endometrial cancers, and reveals that reduced endometrial cancer risk was stated for increased Qu intake, a flavonol [197]. The flavonoids, including Qu, can repress cancerous cell metastasis via reducing expression of c-Myc [143], which have been reported to induce EMT signaling in endometrial cancer [198]. In a case control study conducted by Rossi et al. in Italy, which investigated proanthocyanidins and other flavonoids in relation to endometrial cancer risk, no association was found for other classes of flavonoids with an exception to Qu, which showed inhibition effects on endometrial cancer conditions [199].

### 3.5. Pancreatic Cancer

Pancreatic cancer is the eleventh most common cancer worldwide, accounting for 4.5% of global cancer deaths, and is more common in men than women [1]. Several risk factors have been associated with intractable malignancy; however, smoking, alcohol abuse, age, and family history are most common [200]. Increasing evidence suggests that food-derived polyphenols have a beneficial effect on cancers. Qu has been shown to exert anticancer and anti-inflammatory effects through the inhibition of several intracellular pathways [42] and inhibits several key signaling components in cancer cells. By inducing apoptosis, autophagy, and cell cycle arrest, blocking cell migration, and inhibiting fatty acid synthesis required for de novo membrane synthesis, Qu elicits antitumor effects [201,202,203,204,205]. More recently, Qu has been shown to inhibit self-renewal capacity of putative pancreatic cancer stem cells [144]. Several in vivo animal models indicate suppressed local and distant tumor growth post administration of Qu in pancreatic cancer cases. Mouria et al., through a nude mouse model, stated that in vitro Qu and trans-resveratrol, significantly increased apoptosis, causing mitochondrial depolarization and release of cytochrome c, followed by activation of caspase 3 and apoptosis [206]. Since the bioavailability and intestinal absorption of orally administered polyphenolic compounds, e.g., flavonoids are often limited.

Engst et al. conducted an in vivo nude mouse model and combination study for Qu, alone and in combination with gemcitabine, and concluded that Qu robustly induced cell death, leading to a decrease in cell growth in pancreatic cancer cells in vitro, and if given orally, could significantly attenuated tumor growth in vivo. Results demonstrate that the growth inhibitory effects of Qu are mediated by an increase in apoptotic cell death. It also gave first evidence that dietary supplementation of Qu is effective at inhibiting pancreatic cancer growth in vivo, which was initially limited to animal models [145]. Another study, conducted by Pang et al., concludes that Qu might target cluster of differentiation (CD)36 and decrease the death rate caused by pancreatic cancer by increasing cell adhesion, facilitating fatty acids uptake, regulating thrombospondin 1, and increasing immune response [146]. Fan et al. demonstrated non-development of resistance in pancreatic cancer cells to natural chemopreventers, such as sulforaphane or Qu, as induced in chemotherapy. The constant exposure of pancreatic cancerous cells does not stimulate resistance in living cells, but decreases tumorigenicity by inhibiting tumor progression markers [207]. 

Numerous animal and laboratory research indicates that sulforaphane and Qu prevent metastasis and proliferation, increase apoptosis, and eliminate cancer stem cell characteristics in pancreatic cancer. Hence, these bioactive agents are believed as potential future therapy options [42,208].

### 3.6. Gastric Cancer

Gastric cancer (GC) is the second leading cause of cancer-related deaths, and nearly 760,000 incidents of stomach cancer are diagnosed around the globe [209]. Qu not only has potent antioxidant properties via free radical scavenging [210], but also decreases inflammation and prevents angiogenesis and cell proliferation, due to which it can prove beneficial in the treatment of gastric cancer [211].

A case-controlled study conducted by Ekstrom et al. demonstrated that a diet with high total antioxidant capacity was linked with a decreased risk of gastric cancer, and strong reverse links between Qu and the risk of non-cardiac gastric adenocarcinoma [212]. Qu can also prevent procarcinogens activation by modulating the cytochrome P450 enzymes expression. Apart from antioxidative properties, the role of Qu in the inhibition of ROS and intervention in signaling pathways is particularly important, with respect to gastric cancer. ROS remains relatively harmless, but when produced excessively, it disrupts antioxidant balance, leading to toxic metabolites, which may lead to the initiation and promotion of cancer [213]. Qu can decrease high valent iron to prevent lipid oxidation and reduce production of iron-catalyzed ROS [77]. As an outstanding antioxidant in vitro, its antioxidant activities are mostly exhibited by influencing ROS, glutathione, and enzyme activity, and regulating signaling transduction pathways [74]. As an efficient ROS scavenger, Qu utilizes the antioxidant effects by free radical scavenging activity, decreases the level of ROS, and prevents lipid peroxidation [42,76]. Qu is also considered to induce LC3 turnover and initiate autophagy-related genes in gastric cancerous cells. The lipidated form of LC3 converting from LC3-I to LC3-II has been deemed an autophagosomal marker due to its aggregation and localization on autophagosomes [147]. Inhibition of autophagy enhanced Qu-induced apoptosis in gastric cancer cells were augmented when combined with chloroquine, leading to overall suppression of gastric cancer cell growth [205]. Qu prevented the Akt-mTOR signaling pathway in gastric cancerous cells. The Akt-mTOR signaling is deemed as a vital negative regulator of autophagy [214].

Li et al., in his study, suggested Qu as a promising agent against GC metastasis, since it exhibits antimetastatic effects on GC cells [148]. Previous study reported the effects of quercetin on the apoptosis of the human gastric carcinoma cells [215]. Another study conducted by Shen et al. revealed that Qu causes mitochondrial apoptotic dependent growth inhibition through the blockade of PI3K/Akt signaling in gastric cancerous stem cells, representing a potential target for the therapy of gastric cancer, since they inhibit the growth of cancer stem cells [92]. Kim et al. highlight that, during the intrinsic pathway apoptosis, Qu activates caspase-3, -8, -9, encourages the Bad, Bax expression, and downregulates the antiapoptotic proteins, which can affect tumor development by regulating epigenetics. It regulates the miRNA expression and DNA methylation level to utilize anticancer effect, and increases the tumor cells sensitivity to chemotherapy [216]. In an in vitro study conducted by Zhang et al., combined treatment with curcumin and Qu was experimented with, results conclude substantial inhibition of cell proliferation, accompanied by reduced phosphorylation of AkT and ERK, loss of mitochondrial membrane potential, and cytochrome c release. These outcomes suggest that the combination of Qu–curcumin stimulates apoptosis via the mitochondrial pathway. Conspicuously, the effect of combined treatment with Qu–curcumin on gastric cancer cells is stronger than that of single treatment, suggesting that Qu–curcumin combinations have potential as anti-gastric cancer drugs for more development [217]. Recently, the Network Pharmacology Approach was used by Yang for systematic elucidation of the mechanism and target pathways of Qu in treating gastric cancer. Furthermore, the application of Qu in gastric cancer treatment has been reported [218].

### 3.7. Liver Cancer

Liver cancer or hepatocellular carcinoma (HCC) is one of the most common malignant tumors and top causes of cancer-induced deaths worldwide. HCC is an extremely common primary liver tumor, with cholangiocarcinoma being the second most common. Due to such high complexity, it is difficult to develop a successful therapeutic platform. 

Flavonoids, such as Qu, possess therapeutic anticancer functions, and therefore may prove beneficial in the treatment of hepatocellular carcinoma [219,220]. Accumulative evidences from previous studies show that Qu might protect the liver from concanavalin A-induced fibrosis, acute hepatitis, and ischemia reperfusion injury [221,222,223]. Others have stated that Qu had efficient anticancer functions in HCC [149] and this act may have a close connection with the STAT3 pathway [115,224]. As a cytokine, STAT3 has indicated that it is strongly linked with development of tumors. STAT3 can prevent autophagy and apoptosis [225,226] and promote invasion and migration of primary cancers, such as HCC. 

Research by Wu et al. shows Qu could suppress HCC proliferation and induce apoptosis [150]. Moreover, it supports autophagy of HCC. These effects were related with the JAK2/STAT3 signaling pathway, which were potentially inhibited by Qu, hence reducing the progression of liver cancer. The research provides evidence for the antitumor function of Qu, which could turn into potential therapy for patients with liver cancer [150]. In another clinical study, conducted by Brito et al., patients in advanced stages of HCC were treated with Qu, and the possible synergetic effects between Qu and sorafenib (a nonspecific multikinase—an inhibitor utilized in clinical practice) were assessed. It was observed that, in all cell lines, Qu caused inhibition of the metabolic activities and cell death by apoptosis, followed by a rise in ratio of BAX/BCL-2 [227]. 

Another in vitro study, conducted by Hisaka et al., stated that Qu prevents the liver cancerous cell proliferation through initiation of apoptosis and cell cycle arrest. Liver cell lines were treated with different dosages of varying concentrations of Qu, and cell viability along with cell cycle progression, were assessed, which concluded that treatment with Qu and 5-Fuorouracil caused a synergetic effect, and most cell lines showed cell cycle arrest at different cell cycle stages [151]. Qu regulates other related signaling pathways via initiation of the phosphorylation of Sp-1, Src-1, and STAT5, which are responsible for the progression of cancer [228]. 

### 3.8. Colon Cancer

Colon cancer is one of the most common cancers prevalent around the globe, but mainly in western nations. The high incidences are often associated with a western style diet and meat-dominant dietary consumption patterns [229]. Qu can prevent cancerous cells growth with the capability to act as a chemopreventer.

Zhang et al. demonstrated that Qu induces apoptosis in human colon cancerous cells via inhibiting the NF-*κ*B pathway, upregulation of Bax, and downregulation of Bcl-2; hence, providing a basis for clinical applications of Qu in colon cancer incidents. The research showed that Qu presented effective anticancer effects in an inhibitory effect on NF-κB, and can cause colon cancerous cells apoptosis in vitro [230]. Another study, conducted by Yang et al., concluded that Qu affects HT-29 human colorectal cancerous cell apoptosis, cell cycle arrest, and viability. The overexpression of CSN6 decreased the effect of Qu treatment on HT-29 cells, indicating that Qu induced apoptosis [152].

During the process of apoptosis, TRAIL plays a positive role since it is capable of initiating apoptosis through engagement of its death receptors. Qu affects the expression of TNFR1, TRAILR, FAS, etc. [65]. It can collaborate with TRAIL to stimulate apoptosis in NSCLC, glioma, colon cancer, and several other cancerous cells through two-ways, increasing the DR5 expression and preventing the expression of surviving [93,94]. In a study conducted by Priego et al., Qu has been demonstrated to enhance chemo-radiosensitivity against colorectal cancer in xenograft mouse models; hence, being a potential supplementary therapy option.

### 3.9. Renal Cancer

Clear cell renal cell carcinoma (ccRCC) is the most widespread malignant tumor in the kidney, and more than 20 percent of patients—even after therapeutic surgery—develop severe metastasis during follow-up [231,232]. Aggressive tumor cell migration and invasion leads to difficulty in the development of preventive techniques with limited therapeutic options. Therefore, antitumoral effects of a potential chemopreventive natural product, such as Qu, are being explored immensely with respect to renal cancer prevention.

Meng et al. performed a study based on Qu, combined with the antisense oligo gene therapy, and reported that either Qu or oligo gene therapy play an excellent role in repressing cell migration, proliferation, and induction of apoptosis. The combination of both provide strong repressive effects towards cancer cells. This study provided the chance of using a novel therapy for renal cancer, by combining gene therapy and natural products [233]. The study by Buonerba et al. emphasized the role of Qu in mitigating the adverse impact of sunitinib (a primary renal cancer treating drug). It was observed that Qu-3-*O*-β-d-glucopyranoside has an enhanced pharmacokinetic report. On the grounds of the in vitro stimulatory activity, with respect to AMPk, oral administration of Qu could improve fatigue in kidney cancer patients receiving sunitinib [153]. Another combination drug therapy study conducted by Wei Li et al. asserts that, since Qu and hyperoside (QH), in combination of a ratio of 1:1, have earlier demonstrated to prevent the growth of human leukemia cells; the same can be replicated by examining the anticancer activities of the same mixture in 786-O renal cancerous cells. It was concluded that QH decreased the generation of reactive oxygen species [234]. A recent study suggests that Epigallocatechin gallate (EGCG) may play a preventative role in renal cell carcinoma development [235]. This study assessed the effect of TRAIL, EGCG, and a combination of both on a TRAIL resistant renal cell carcinoma cell line. The information shows that EGCG alone offered a substantial decrease in cell viability, but co-therapy with TRAIL provided a noticeable decrease in cell viability more than that of TRAIL or EGCG alone. Qu has been described to increase EGCG activity in terms of bioavailability in animal models [236]. The impact of Qu on kidney fibrosis, which is significant in renal cancer, have been studied by Ren, et al. through a mice model. The study indicated Qu administration could largely improve kidney interstitial fibrosis and macrophage accumulation in the kidneys with obstructive nephropathy. It concluded that Qu could potentially reduce fibroblast activation and kidney fibrosis, including repression of the mammalian target of rapamycin and β-catenin signaling [237].

### 3.10. Prostate Cancer

Prostate cancer is a common male malignant disorder, and its prevalence is increasing worldwide. Non-promising results of the existing treatment therapies call for alternative medication. In recent years, the chemopreventive effects of Qu on prostate cancer have attracted the attention of researchers, and were shown in animal experimentations [131,238,239]. Earlier researchers have observed that Qu provokes prostate cancer by decreasing expression of androgen receptor (AR), by causing apoptosis and repressing proliferation [154]. Recently, several studies have demonstrated the anticancer properties of Qu in numerous human cancerous cell lines, both in vitro and in vivo, including prostate cancer [240]. Through studies, Qu’s effect on the proliferation of human prostate cancerous cells treated with varying dosages has been investigated and it was observed that the rate of inhibition showed a dosage-dependent increase. Treatment of Qu not only resulted in a rise in the G2-M phase population in both LNCaP and PC-3 cells, but also improved the S-phase population in PC-3 cells [238]. Liu et al. treated PC-3 cells with Qu and reported that cell viability was substantially reduced in a time- and dosage-dependent manner [241]. Treatment of Qu decreased AR expression and then increased caspase-3 and -7, initiating subsequent apoptosis and anti-proliferation in LNCaP cells [242]. In a study conducted by Asea et al., Qu, at 150 mg per kg, was administered intraperitoneally in DU-145 and PC-3 xenograft tumor models; it repressed growth of the xenograft tumor ascribed to the antagonization of expression of HSP72 [111]. In vitro studies have confirmed that Qu efficiently prevents the growth of several prostate cancerous cell lines. When utilized in vivo, Qu also prevents growth of the prostate cancer xenograft tumor. Epidemiological studies suggest that a Qu-rich diet results in a lower risk of prostate cancer, and certain in vivo researches in animals have confirmed the chemopreventive effects of Qu in prostate cancer. The pharmacokinetics of Qu has been studied in humans and a clinical case-control study demonstrated that Qu decreased prostate cancer risk [68].

### 3.11. Urinary Bladder Cancer 

One of the most evident carcinomas of the urinary tract is bladder cancer [243]. One of the most common standard treatments is intravesical chemotherapy; however, recurrence and metastasis cause problems in providing a suitable cure [244]. In addition to existing therapies, novel treatments, such as targeted therapy, has made significant improvement in cancer treatment [245]. A recent study by Su et al. on the two human—and one murine—bladder cancer cell lines, to observe the killing effects of Qu and the underlying processes, exhibited the antiproliferative potential of Qu on cancerous cells by activating the AMPK-signaling pathway, inducing apoptosis and inhibiting migration, providing a solid basis on the clinical applications of Qu in treatment of bladder cancer. The evaluation of colony suppression of Qu on the bladder cancer cell line have also been conducted; it was assessed that, at 80 µM concentrations, colony numbers were considerably reduced at three bladder cancerous cells, and the most sensitive cell line, which is constant with MTT, 3-(4,5-dimethylthiazol-2-yl)-2,5-diphenyltetrazolium bromide experiment results, stating overall ability of Qu to inhibit bladder cancer cell growth. Moreover, it was observed that Qu induced apoptosis in the bladder cancer cell line, depending upon the administration of dosage [155]. Te-Fu Tsai et al. performed a study based on bladder cancer and concluded that Qu pre-treatment against autophagy inhibitors, Baf A1, and chloroquine, strongly augmented apoptosis, suggesting the repression of Qu-induced autophagy increased apoptosis. Therefore, a combined treatment of inhibitors of autophagy, which induces cells to Qu treatment, may be a better therapeutic method to decrease proliferation of bladder cancerous cells [246]. Another study examined the cytotoxic effect of Qu against T24 cells, analyzed by an MTT experiment, clonogenic assay, and DNA-damaging effect by comet assay. The study showed a high connection between a decreased number of colony and viability of cells and an increase in DNA-damage of T24 cells incubated with Qu during short-term incubation (2 h). The research indicates that Qu could prevent proliferation of cells and formation of colony human bladder cancerous cells by causing DNA damage, and that Qu may be an efficient chemopreventive and chemotherapeutical agent for papillary urothelial bladder cancer after transurethral resection [156].

### 3.12. Leukemia

Acute myelogenous leukemia (AML) is a destructive disorder with high mortality and morbidity [247], and is characterized by bone marrow failure, clonal proliferation, and an enhanced risk of acute myelogenous leukemia development [248]. Traditionally, leukemia was deemed to be the result of genetic changes; however, in recent time, it has been demonstrated that it may also prevail due to stimuli-triggered changes in gene expression (epigenetic alterations) [249,250]. Studies from the past show that Qu has repressed the proliferation of cancers, including leukemia [103]. Accumulative studies suggested that Qu synergistically sensitizes to apoptosis numerous leukemic cell lines and B-cells isolated from patients of Chronic lymphocytic leukemia (CLL) [157]. Naimi et al. also studied the synergist effects of TRAIL and Qu in human myeloid leukemia cells, and demonstrated that Qu can be utilized as a warning factor along with TRAIL, supporting the influence of TRAIL-induced apoptosis [158]. 

Calgarotto et al. examined the in vivo antitumor efficiency of Qu and green tea in human leukemia. Green tea and Qu decreased growth of tumor in HL-60 xenografts escorted by reduced antiapoptotic protein expression and enhanced BAX expression, a proapoptotic protein. Furthermore, caspase 3 was stimulated to a larger extent after green tea and Qu treatment. Green tea and Qu also facilitated in the cell cycle arrest of the G1-phase in HL-60 xenograft models and were able to initiate the autophagic progression [251]. An in vivo investigation was conducted by Alvarez, et al. to understand the molecular processes underlying the proapoptotic effects of Qu by assessing the Qu therapy effect on posttranslational histone modification and DNA methylation of genes related to the apoptotic pathway. The findings demonstrated that increased apoptosis, caused by Qu, might be caused in part by its DNA demethylating activity [252]. Qu efficiently causes cell death in human leukemia cells in vitro, and in leukemia xenograft models, and that phenomenon stems from a method involving a multilevel of cooperation among cell cycle arrest, apoptosis, and autophagy. The antitumor activity of Qu both in vitro and in vivo, point to Qu as an attractive antitumor agent for hematologic malignancies [253].

### 3.13. Lymphoma

A heterogeneous group of cancers, which arises mainly from lymphoid tissue all over the human body, is called non-Hodgkin’s lymphoma (NHL) [254]. It is reported to be caused mainly by risk associated with immunosuppression and infections, but the causes remain unclear. It is a common type of cancer among both men and women [255]. 

The effects of Qu and cyclopamine on the in vitro growth and expression of protein of B-cell lymphoma cell lines were examined. Qu reduced Gli1 protein level, a target gene product of Hh signaling, and repressed the lymphoma cell growth. This result indicates the potential usage of these compounds in molecular targeted treatment for lymphoma [256]. Qu also decreased the growth and viability of B-lymphoma (PEL, an aggressive B cell lymphoma cell) cells BC1, BC3, and BCBL1 in a broad variety of concentrations, but had no cytotoxic effect in normal B-lymphocytes [224]. Molecular processes of TRAIL resistance and reactivation of the apoptotic machinery by Qu in non-Hodgkin’s lymphoma (NHL) cell lines were determined by Jacquemin et al., where the role of Qu in mediating survivin and Mcl-1 downregulation restoring TRAIL-induced apoptosis in NHL B cells was assessed. The findings show that Qu restores TRAIL induced cell death in resistant transformed follicular lymphoma. Qu rescues mitochondrial activation via causing the proteasomal degradation of Mcl-1 and by preventing expression of surviving [159]. Several studies observed that Nf-*κ*B is a crucial inflammatory modulator for Qu to kill cancerous cells, including lung and melanoma cancer [257]. OMe-Qu analogs have been demonstrated to prevent proliferation of cells after 72 h incubation with human breast, neck and head, melanoma, lung, and cervical cancer cells [258]. Qu’s effect on the metabolism of glucose has also been shown in vivo via a mice model, and is related to its ability to modulate the PI3K-Akt pathway. In a dosage-dependent manner, the cell viability of the ascites cells of the lymphoma mice were examined without causing liver toxicity. Reduced Akt level, an important mediator of PI3K signaling, and p85α phosphorylation, an essential component in activation of PI3K, changed PI3K signaling in Dalton’s lymphoma mice treated with Qu [259]. High intake of flavonoids, dietary elements with numerous putative anticarcinogenic activities, may be linked with lower risk of NHL [260]. 

### 3.14. Lung Cancer

Lung cancer is one of the most commonly diagnosed cancers, globally, with an average survival rate of 5 years in most countries [261]. It is one of the leading worldwide causes of death related to carcinoma, with nearly 18% affecting both men and women. It is primarily of two types—non-small cell lung cancer, which comprises of 85% of lung cancers, and adenocarcinomas [262]. Major risk factors associated with lung cancer are smoking tobacco, exposure to chemical carcinogens, and harmful inflammatory diet (high salt consumption, low consumption of vegetables and fruits, etc.) [263]. By far, the prognosis remains unsatisfactory, despite the availability of traditional treatment options involving surgery, radiation, chemotherapy, and specific targeted therapies, primarily due to late diagnosis and late appearance of metastasis, especially in conditions associated with lung cancer [264,265]. The role of numerous flavonoids in lung cancer have been discussed by Zanoaga et al. and Collins et al. [266,267], and Qu, identified as having antitumor properties, has been tested for its impact on aurora B activities in a study conducted by Xingyu et al. [160]. Aurora B, a protein part of series of serine–threonine kinase, is important during attachment of mitotic spindle to the cancer cells and centromere; overexpression of aurora B causes uneven division of genetic information, generating aneuploidy in cells, a characteristic of cancer [268]. Several inhibitors of aurora B have several side effects, such as anti-proliferation toxicity on the bone marrow, anemia, neutropenia, dry skin, alopecia, etc. [269,270]. Qu has also been identified as an antitumor agent, as observed by Xingyu et al., who concluded that Qu in in vitro and in vivo conditions inhibited aurora B activities by directly binding with aurora B. Ex vivo studies showed that Qu inhibited aurora B activity lung cancer cells. An in vivo study showed that Qu injection in A549 tumor-bearing mice efficiently repressed growth of cancer [160]. Generally, mostly studies on Qu concentrated on its growth inhibitory action through initiation of apoptosis [65] or autophagy [271] development of chemosensitivity [272] and Qu exerted the anti-NSCLC effect by inhibiting Src-mediated Fn14/NF-*κ*B pathway both in vitro and in vivo [273].

However, more recent work also focuses on the effects of Qu on tumor cell metastasis and invasion. In lung cancer conditions, metastasis is considered as the major cause of death. A study conducted on snails/animals found that Qu inhibited the migration or invasion of NSCLC cell lines and bone metastasis in an orthotopic A549 xenograft model, by repressing the snail-mediated EMT, and the survival time of animal models were also extended after Qu therapy. The study clearly showed that Qu could substantially prevent tumor bone metastasis and extend the lifespan in a human A549 xenograft model, suggesting that Qu has potential value in clinical applications for NSCLC [107]. Anticancer effects of Qu and its underlying molecular mechanisms in non-small cell lung cancer cells have also been studied by Youn et al., wherein Qu was observed to enhance the gene expression linked with death receptor signaling and inhibition of the cell cycle, but reduce the expression of genes involved in activation of Nf-*κ*B. Qu was observed to induce apoptosis by inhibiting nuclear factor-kappa B signaling in H460 lung cancer cells, proving its usefulness in its prevention [65].

### 3.15. Osteosarcoma

Osteosarcoma is a kind of cancer of the bone, which starts in the cells that are involved in the formation of bones. It is extremely metastatic, infects bone and soft tissues frequently, and locally proliferates to the lung. It occurs, most commonly, in the long bones of the body (e.g., the leg bones), and can begin sometimes in the arms, but it seldom occurs in soft tissue outside the bone. It generally occurs in young adults and adolescents, but it can also occur in older adults [274]. Lan et al. reported that Qu considerably attenuated invasion and migration of osteosarcoma cells in comparison to treatment with a control medium, and may have potential in human osteosarcoma therapy [161]. Li et al. assessed the effects of Qu-induced inhibition of parathyroid hormone receptor-1 on invasion, proliferation, and migration in cancer cells. It stated that Qu significantly reduced the mRNA expression levels of PTHR1 and enhanced Qu-inhibited proliferation and invasion, thus reducing human metastatic osteosarcoma cell invasion [162].

### 3.16. Brain Tumor

Glioblastoma (GB) or glioblastoma multiforme (GBM) is the most destructive and common form of brain tumor, which is a malignant tumor of connective tissue [275,276]. Presently, development of a tumor drug has switched from traditional cytotoxic drugs to the agents, which aim the specific molecules in which flavonoids have turned to be quite effective interventions in various stages, processes, and phases of different cancer progression. In case of lung cancer, flavonoids have been observed to stimulate microglia and subsequently attenuate tumor cell migration, stimulate immune responses, and decrease proliferation of glioma cell, along with preventing the growth factor expressions in cultures.

Qu is a potent anticancer agent for the treatment of brain tumors. Numerous researches have stated that Qu enhances the efficiency of glioblastoma therapy by repressing the PI3K-Akt pathway [163], arresting cells at the G2 checkpoint of the cell cycle, and reducing the mitotic index in glioma cells [277], blocking STAT3 [115], and causing apoptosis by reducing the X-linked inhibitor of the apoptosis protein (XIAP) [278] [278]. Multiple lines of data have demonstrated that Qu regulates many proteins involved in the cellular signal transduction in GB [279]. A study by Kiekow et al. in 2016 concluded that Qu was observed to effectively prevent the growth of GB cells and stimulate apoptosis by repressing the PI3K/AkT, NF-*κ*B, and Ras/MAPK/ERK signaling pathways [164,280]. Siegelin et al. have shown that combining Qu with tumor necrosis factor-related apoptosis-inducing ligand (TRAIL) significantly increased TRAIL-mediated apoptosis in U87, A172, U251, and LN229 GB cells, signifying that Qu sensitizes GB cells to death-receptor mediated apoptosis by suppressing the inhibitor of apoptotic protein [191]. Santos et al. (2015) showed that Qu slowed down cell migration in human GL-15 GB cells, likely by decreasing the expression of metalloproteinase, MMP2, and by increasing the laminin and fibronectin expression [281]. Findings by Liu et al. showed that low concentration of Qu antagonized GB cell invasion and angiogenesis in vitro. Qu inhibited GB cell migration and angiogenesis by downregulating the expression of VEGFA, MMP2, and MMP9 [282]. In molecular terms, it has also been demonstrated that Qu relates with several proteins, such as VEGF, Bcl-2, PI3K/Akt/mTOR, IL-6, Bax, MMP-2/-9, STAT3, and HSP, which are implicated in GB cell development and signal transduction pathways [279]. 

### 3.17. Head and Neck Cancer 

The EGFR gene is frequently amplified in head and neck squamous cell carcinoma (HNSCC). It is often linked with a bad prognosis and low survival rate. Therefore, efficient inhibition of the EGFR signaling-mediated malignancy would reduce cancer proliferation and improve the survival rate [283]. In order to investigate the impact of Qu as an efficient anticancer in head and neck cancer, a study was conducted by Chan et al., which supported the possibility of Qu as an effective anticancer agent in EGFR, overexpressing HNSCC. Qu effectively prevents the cellular invasion and migration of the HNSCC cell lines, FaDu, and HSC-3. It can be taken as potentially another treatment for HNSCC patients, carrying an abnormal EGFR signaling axis, which suppresses cellular invasion and migration in HNSCC [165]. The effect of Qu on the in vitro and in vivo growth of a normal human lung fibroblast-like cell line, and two squamous cell carcinoma cell lines, was studied by Castillo et al.; it was observed that Qu seems to have a cytotoxic effect on squamous cell carcinoma of head and neck origin, both in vivo and in vitro [166]. 

### 3.18. Oral Cancer

Oral cancer is a cause of cancer-associated mortality around the globe. The traditional therapy modalities of oral cancer involve chemotherapy, radiation, and surgery. The role of flavonoids, including quercine, have evolved as potential chemopreventers in the recent past. Several studies have been conducted in recent decades to study the role of quercine in the prevention of oral cancer. In a study conducted by Ma et al., Qu encouraged apoptosis of human oral cancer (SAS) cells via mitochondrial and ER mediated signaling pathways [106]. A similar study conducted by Lai et al. reflected that Qu prevents invasion and migration of SAS human oral cancer cells via inhibition of matrix metalloproteinase-2/-9 and NF-*κ*B signaling pathways [167]. Qu significantly prevents cell growth and migration, and invasion of oral cancer cells. The molecular and cellular mechanisms involve the cell cycle arrest accompanied by mitochondria mediated apoptosis. The outcomes indicate that Qu may have potential as a new chemopreventive agent, or act as a therapeutic adjuvant for oral squamous cell carcinoma [284].

### 3.19. Esophageal Cancer

Esophageal cancer (EC) is considered the sixth highest contributor to all cancer-related mortality around the globe. Despite advancement in EC treatment, presently used methods remain ineffective [285]. Consumption of fruits and vegetables have been associated with decreased several cancer risks, such as esophageal cancer [29,286]. Qu might be a crucial supplement for the management, prevention, and treatment of EC, due to its natural origin. The inhibitory effect of Qu-3-*O*-β-d-glucuronopyranoside on gastritis and reflux esophagitis in rats have been studied by Min et al. and findings demonstrate that Qu-3-*O*-β-d-glucuronopyranoside can prevent gastritis and reflux esophagitis in rats [168]. In a case control study conducted by Lin et al. on a Swedish population with esophageal adenocarcinoma (OAC), esophageal squamous-cell carcinoma (OSCC), gastroesophageal junctional adenocarcinoma (JAC)—the impact of dietary flavonoids, such as lignans, Qu, and resveratrol, to inhibit esophageal cancer, were investigated. It was signified that a dietary pattern characterized by the intake of resveratrol, lignans, and Qu may play a protective role in esophageal cancer development in Swedish people [57].

### 3.20. Melanoma

Melanoma is an aggressive form of skin cancer, characterized as a malignant tumor, it often originates from pigment-producing melanocytes. Owing to its metastatic potential, melanoma is the prominent cause of death in all skin cancer types, generally induced from neural crest-derived melanocytes. Its mortality, incidence, and prevalence has grown significantly over the last thirty years. Dietary compounds, including Qu as anticancer agents, have earned attention due to the latest interpretation of their mechanisms of action. Numerous pathways are proposed to induce melanoma development, such as the Notch, RAS/RAF/MAPK, and PI3K/AKT pathways. These researches have demonstrated a selective sensitivity of melanoma tumor cells to the cytotoxic effects of Qu [287], whereas healthy tissues could be protected via antioxidant activity of the Qu and subsequent initiation of protective cellular signaling pathways [288]. 

Qu is quickly metabolized by tyrosinase into several compounds, which encourage anticancer activity. Furthermore, given that expression of tyrosinase increases during tumorigenesis, and its activity is linked with pigmentation modifications in both early- and late-stage melanocytic lesions, it suggests that Qu can be used to target melanoma [169].

As for Qu’s role in inducing tyrosinase and stress response protein in melanocytic cells—Qu stimulates the tyrosinase expression and numerous stress responsive proteins, including p53 and NQO1.

### 3.21. Myeloma

Multiple myeloma (MM) is a hematological malignancy with plasma cell proliferative disorder. In several in vitro studies, the impact of Qu on myeloma have been studied in the recent past [289]. A study conducted by Ma et al. observed that Qu repressed MM cell proliferation by downregulating the IQ motif containing GTPase-activating protein-1 expression, and the activation of extracellular signal regulated kinase [290]. Moreover, Qu encouraged apoptosis of myeloma cells and showed a synergetic effect with dexamethasone in vitro and in vivo xenograft models, which were studied by He et al. It also revealed that Qu inhibited MM cells proliferation (MM.1R, RPMI8226 and ARP-1) by encouraging apoptosis and the G2/M phase cell cycle arrest; it was concluded that Qu, alone or in combination with dexamethasone, may be an effective therapy for MM [105].

### 3.22. Retinoblastoma

Retinoblastoma (RB) is an eye cancer that starts in the retina, i.e., sensitive lining inside the eye, and is characterized by abnormal appearance of the pupil and leukocoria. RB has been described as being one of the most happening malignancies of an intraocular cavity affecting small children. One in every 15,000 children are reported to be affected by it worldwide [291].

Presently, therapy modalities for RB involve systemic focal treatments, chemotherapy, enucleation, subconjunctival, or intra-arterial chemotherapy and external beam radiation therapy [292]. However, prognosis of these treatments depends on the stage of RB. To enhance the efficiency of treatment, comprehensive knowledge of the mechanism involved in the RB development and oncogenesis is important. In cells of RB, the expression status of several genes is significantly different. These differentially expressed genes involve components involved in the signaling pathways, angiogenesis, immune system, cell proliferation, and structure [293]. Among the latent signaling transductions included in the RB genesis, angiogenesis has been shown to be linked with local metastasis and invasive growth of this cancer [294]. As an alternative therapy for treating RB Qu has been observed to reflect positive benefits. Several in vivo and in vitro researches have shown that Qu can utilize antitumor effects by inhibiting metastasis progression, cell proliferation, and angiogenesis, modifying cell cycle progression, encouraging apoptosis, and influencing autophagy, including retinoblastoma [15,295]. Among all of the signaling pathways involved in the oncogenesis of RB, the invasive nature and spread of cancer is attributed to the process of angiogenesis in this type of cancer type [294]. In addition, a previous study showed tumor angiogenesis as a prognostic component for disease dissemination of RB, which verified the important role of angiogenesis in RB carcinogenesis [170]. Hence, concatenated use of traditional treatments and anti-angiogenic agents may be synergetic for a greater alleviation of the RB impairment [296]. Evidence suggests that Qu inhibited VEGF-related angiogenesis in several tumorigenic cell lines, including retinoblastoma. Qu hindered growth of RB and in vitro invasion in a dosage dependent manner. Additionally, Qu prevented angiogenesis in RB by aiming VEGF. Hence, Qu may be a potential anti-RB therapy based on its anti-angiogenic effect [297]. Another study, conducted by Liu et al. to identify antitumor effects of Qu on retinoblastoma cells, suggest that QCT effects Y79 retinoblastoma cells via activation of JNK and p38 MAPK pathways, causing collapse of mitochondrial membrane potential in Y79 cells [298]. Because anti-cancerous and anti-inflammatory properties of Qu possess an important role by regulating cell cycle, by modulating several molecular targets, including p21, cell cycle arrest occurs at the G1-phase by p21 initiation, and by associated reduction of phosphorylation of the RB protein (pRb) [299]. Similarly, earlier potential impact of Qu in blocking the cell cycle division at the G2/M checkpoint was studied by Ong et al., which concluded that Qu increased RB gene expression in nasopharyngeal carcinoma cells, CNE2 and HK1, and prevented cell cycle in the G2-M phase [300]. 

## 4. Bioavailability of Qu

Bioavailability is the capability of a drug or other element to be absorbed and utilized by the body. The small part of substances that are orally administered are absorbed and available for physiological activities or storage [301]. From a nutritional perspective, it can also be characterized as the level of absorption, digestion, excretion, and metabolism of a compound after food intake [302]. Generally, when substances or drugs are consumed orally, dietary polyphenols, such as Qu, are primarily absorbed and metabolized in the small intestine [303,304,305], and only a small proportion of it is absorbed in the stomach [306]. The resulting compounds are further circulated by blood as conjugates with glucuronide, methyl, or sulfate groups attached, used by the body, and eventually excreted in the urine based on its availability. Qu is usually present as Qu-glycoside and includes Qu aglycone conjugate to sugar moieties, such as glucose, and based on the pharmacokinetics assessment, classified as absolute or relative bioavailability [307]. Mostly, Qu found in plants are bound to sugar moieties rather than in free form. The attachments and types of sugar influence the bioavailability and concerned bioactivity. Differences in Qu-conjugated glycosides also affect its bioavailability because the size and the polarities of these compounds cause a hindrance in crossing the membranes [308,309]. In plants, Qu is found attached to sugars, but the absorption unit of Qu is aglycone; therefore, sugar must be removed, which is achieved through enzymes, such as lactase phloridzin hydrolase (LPH), which removes glucose compounds from flavonol [310]. The aglycone is highly reactive, but relatively insoluble in aqueous media (especially lumen of the gut) [311,312] in comparison to Qu glycosides, which are more bioavailable since they are absorbed more quickly than other types of glycosides. They can only be de-glycosylated to Qu aglycone by enzymes from gut flora [313].

Different natural sources from where Qu is derived also seem to influence the bioavailability of this compound [314]. Current evidence also indicates other potential natural sources, such as tea, rich in kaempferol glucoside and retinoid, were the highly bioavailable types. When immersed, Qu and kaempferol are quickly processed in the liver to form sulfate, methyl, and glucuronide metabolites, which can be found in urine and blood [315,316]. Thus, it has been observed that occurrence of sugar-moieties enhances bioavailability and variations in Qu-conjugated glycosides, impacting bioavailability. For example, onion-derived Qu, which is mostly Qu-glucoside, is more bioavailable than apple-derived Qu, which contains Qu-rhamnoside and Qu-galactoside. Qu is a lipophilic compound; therefore, fat increases its bioavailability. Non-digestible fiber can also enhance bioavailability of Qu [317]. As per numerous bioavailability studies conducted to assess the importance of solubility and absorption of Qu conducted on animals and human experiments, it is apparent that the bioavailability of Qu can be enhanced when administered in combination with different dosages of a high fat diet. Therefore, in addition to natural sources, dietetic fat has been demonstrated to improve Qu-aglycone assimilation from the small intestine [318,319]. 

Experiments in pigs demonstrated that Qu-glucoside, in comparison with Qu-aglycone, had larger bioavailability [320]. The manner in which Qu is absorbed also depends on its chemical structure [321]. Based on the available literature on the bioavailability of Qu, and the extensive study conducted by Almeida et al., on the potential publications pertaining to bioavailability of Qu, out of fifty-five research articles selected on predefined criteria, 35 studies projected Qu absorption by assessing Qu-aglycone after hydrolysis. Twelve studies projected Qu conjugates, and five studies assessed both aglycone and conjugates after hydrolysis in plasma and/or urine [322]. Further, the main pathways of metabolism of Qu have been widely studied and depend upon the conjugating enzymes, which depend upon various factors, such as genetic polymorphisms, environment, food, and even microbiota composition, leading to substantial interindividual variation in absorption and metabolism of Qu between individuals [322]. Several compounds, solutions, and agents have been used along with Qu to enhance its bioavailability. In a study conducted by Quagliariello et al., hyaluronic acid (HA) hydrogels were utilized as a carrier of Qu; it was stated that HA acted as a binder molecule of CD44, which is adequately enhanced in various cancerous cells and associated with tumor progression [323]. Research is also being conducted to improve the oral administration methods of Qu—in order to improve its bioavailability. In a study conducted by (Riva et al. 2019) aiming to relate a new food grade lecithin-based formulation of Qu, Qu-phytosome, to indistinct Qu in terms of solubility in simulated gastrointestinal solutions and oral absorption, in randomized crossover pharmacokinetic research of healthy individuals. Substantial advancements in both in vitro solubility and oral absorption (in both maximum concentration and exposure) attained by healthy individuals in a human clinical study were found with the Qu-phytosome formulation, in comparison with unformulated-Qu. An extra soluble Qu formulation based on lecithin, Qu-phytosome has lately been created, and was observed to mediate the accomplishment of higher Qu plasma levels, up to twenty times more than what was generally found following a dosage of Qu—when the new formulation was orally administered in healthy individuals, and it did not have any distinguished side effects. These findings indicate that Qu-phytosome permits the oral administration of Qu in a safe and bioavailable way, thus assisting the efficient use of this natural compound to cure several human disorders [324]. Another study, conducted by Riva et al., used phytosome to boost the improvement in absorption of Qu where hydrophilic Qu was free from the phytosome, into the lipid environment of the enterocyte membrane, permitting it to enter into the bloodstream. They concluded that phytosomes could be utilized to encourage the solubility of poor bioavailable active natural elements and their capabilities to cross biological blocks, significantly improving the bioavailability and bioactivity of Qu [324]. In vitro research also posed a theory that glucose-moiety uses a transporter that usually pumps glucose across the wall of the intestinal membrane [325]. The bioavailability and health effects of dietary flavonoids were reviewed by Hollman and Katan [326]. They observed that Qu-glycosides from onions were more quickly absorbed than pure-aglycone. Moreover, it was noticed that absorbed Qu eradicated gradually from the bloodstream, which indicates that the enterohepatic circulation may be in effect [309]. 

The recommendation was also made that glycosides may be absorbed through intestinal sugar-uptake; it was suggested that the glucuronic acid effect in trained rats includes decreased tissue peroxidation that acts as an antioxidative defense process and encourages healing of muscle weakness [312,327]. Despite potential benefits of Qu, which makes its bioavailability important, unfortunately, its bioavailability remains low. Several factors have been found to affect the bioavailability of Qu. Its significance in cancer prevention requires further research to evaluate and improve its bioavailability. 

## 5. Conclusions

Utilizing compounds derived from plants, commonly called phytochemicals, have been extensively considered for the treatment of cancer in recent decades. Due to their substantial potential abilities in cancer prevention, including anti-inflammation, antioxidation, anti-proliferation, and cell cycle arrest, phytochemicals including Qu have gained importance as potential complementary and alternative medicine (CAM). This compound is found in different foods and beverages, such as tomatoes, grapes, wine, orange juice, onions, peppers, strawberries, lettuce, etc. 

Several methods and techniques have evolved over the years, to examine the potential abilities of the natural chemopreventers, which have been observed to strengthen the impacts of other chemotherapeutic medications, increase their therapeutic effects, and lower their toxicity. The bioactive compound was shown to significantly affect different cancer pathways, as well as several processes and stages related to it, and have inhibition effects on a wide range of cancers, such as nasopharyngeal, colorectal, breast, pancreatic, kidney, ovarian, prostate, and lung cancers. The research article thus presents a cumulative compendium of extensive researches investigating the potential therapeutic role of Qu, in treatment of various types of cancers. Besides several advantages of Qu, it is also associated with several limitations, primarily related to limited research in several cancer domains, poor absorption, rapid metabolism, chemical instability, rapid systemic elimination, and all-over poor bioavailability. 

Therefore, based on the several therapeutic advantages of the compound highlighted in this paper related to cancer inhibition, it strongly supports the development of clinical trials and the incorporation of new approaches, such as nanotechnology to its application, which can significantly enhance the potential of Qu as a powerful therapeutic agent. It can open new horizons in effective utilization, wider applicability, and better bioavailability of Qu as a potent natural chemopreventer, alone or as a combination drug, for better cancer prevention and management. 

## Figures and Tables

**Table 1:** Major mechanism of action of quercetin in cancer management.

Major Mechanism	Result of the Study	Refs
Inflammation	Quercetin discourages cancer development by hindering the development of inflammation producing enzymes (protein kinase C, cyclooxygenase, and lipoxygenase) and reducing proinflammatory-mediators production.	[59]
Inflammation	Inhibitory effects of Qu associated with prostate cancer have been demonstrated through several clinical studies, where it reduces inflammation and associated risk of prostate cancer.	[68,69]
Reactive Oxygen Species (ROS)	The ROS production in (prostate cancer) prostate cancer cells through its pronounced impact on mitochondrial integrity and its antioxidant has been confirmed by Qu treatment.	[75]
Reactive Oxygen Species (ROS)	Lipid oxidation and iron-catalyzed ROS production is inhibited by Qu treatment.	[77]
Angiogenesis	The antitumor effects of Qu has been explained through its anti-angiogenesis effects.	[80]
Angiogenesis	Suppression of ovarian cancer cell growth by intravenously administering QU/m-PEG-PCL micelles, which, amazingly, inhibit the growth of xenograft ovarian tumors by repressing angiogenesis, which reflect novel application of Qu in treatment of ovarian cancer.	[81]
Apoptosis	Qu is associated with stimulating intrinsic pathways and increases the release of cytochrome c from the mitochondria to the cytoplasm. Both processes are responsible for death of tumor cells and inhibit chances of cancer progression.	[91]
Apoptosis	The induction of apoptosis has been noticed in the Qu treatment gastric cancer stem cell.	[92]
Cell cycle	Qu therapy might lead to G0/G1 cell-cycle arrest in leukemia.	[98,99]
Cell cycle	The anticancer role of Qu is measured through the induction of apoptosis and G2-M cell cycle arrest.	[105]
Phosphatidylinositide-3-kinase (PI3k)/Protein kinase B (PKB) pathways	Qu mediates modulation of the PI3K/Akt signaling pathway and modulates suppression of the PI3/Akt signaling pathway survival signals in inhibition of growth of lymphoma.	[114]
Signal transducer and activator of transcription 3 (STAT3)	The anticancer activity of Qu has been identified through the reduction of glioblastoma cell proliferation and migration via suppressing activation of Signal transducer and activator of transcription 3 (STAT3).	[115,117]
Epidermal Growth Factor Receptor (EGFR)	In the in vitro model, Qu inhibits EGF-induced Epithelial-to-mesenchymal transition through the EGFR/PI3K/AKt/ERK-1/2 pathway and suppresses the transcriptional repressor slug, twist, and snail in prostrate cancerous cell line PC-3. Hence, Qu inhibits metastasis of cancer by aiming EMT.	[131]

**Table 2:** Quercetin role in cancer management through modulating cell signaling pathways.

Types of Cancer	Mechanisms/Results of the Study	Refs.
Cervix cancer	Qu causes cell death in cervix cancer by decreasing O-GlcNAcylation of adenosine monophosphate activated protein kinase.	[139]
Cervix cancer	Qu steadily alters the WNT, PI3K, and MAPK pathways by regulating the expressions of various proteins, which leads to the inhibition of cell cycle arrest, cell proliferation, apoptosis, and DNA damage in cervical cancerous cells.	[140]
Breast cancer	Human breast cancer MDA-MB-231 cell death via mitochondrial- and caspase-3 dependent pathways.	[56]
Breast cancer	Qu targets the VEGFR-2 mediated angiogenesis pathway, suppresses the expression of the downstream regulatory factor AKT, and inhibits growth of tumor.	[80,85]
Ovarian cancer	Qu interferes with intracellular signaling pathway induced apoptosis through mitochondrial intrinsic and caspase dependent pathways.	[141]
Ovarian cancer	The intervention of Qu leads to ER stress, increased expression of Bax, p21, and p53, reduced Bcl-2 expression, continued repair of DNA, and causes radio sensitization in ovarian cancerous cell.	[142]
Endometrial cancer	The flavonoids, including Qu, can repress the cancerous cells metastasis via reducing expression of c-Myc.	[143]
Pancreatic cancer	Self-renewal capability of putative pancreatic cancerous stem cells was inhibited by Qu.	[144]
Pancreatic cancer	Qu, alone and in combination with gemcitabine, robustly induced cell death, leading to a decrease in cell growth in pancreatic cancer cells.	[145]
Pancreatic cancer	Cluster of differentiation (CD)36 is targeted that decreases the death rate caused by pancreatic cancer by increasing cell adhesion, facilitating fatty acids uptake, regulating thrombospondin 1, and increasing immune response.	[146]
Gastric cancer	LC3 turnover induced by Qu and activated autophagy related genes in gastric cancer cells.	[147]
Gastric cancer	Qu reduces AGS and BGC823 cell invasion and migration, accompanied by decreased uPAR and uPA protein expression, blocking signaling pathways, finally repressing GC cells.	[148]
Hepatocellular carcinoma	The anticancer functions of Qu has been noticed in hepatocellular carcinoma.	[149]
Hepatocellular carcinoma	The role of Qu has been noted as it suppress proliferation of HCC and encourage apoptosis. Qu prevented metastasis of LM3 cells by regulating expression of vimentin, N-cadherin, MMP9, and E-cadherin.	[150]
Hepatocellular carcinoma	Qu prevents the liver cancerous cells proliferation through initiation of apoptosis and cell cycle arrest.	[151]
Colon cancer	Induction of apoptosis was noticed in colon cancer through the Qu treatment.	[152]
Renal cancer	Qu mitigates the adverse impact of sunitinib (a primary renal cancer treating drug); Qu-3-*O*-β-d-glucopyranoside was observed—that is, iso-Qu has an enhanced pharmacokinetic report.	[153]
Prostate cancer	Qu provokes prostate cancer by decreasing expression of androgen receptor (AR), by causing apoptosis and by repressing proliferation.	[154]
Prostate cancer	A total of 150 mg per kg was administered intraperitoneally in DU-145 and PC-3 xenograft tumor models; it repressed growth of xenograft tumor ascribed to the antagonization of expression of HSP72.	[111]
Bladder cancer	Dose dependent administration of Qu caused induction of apoptosis.	[155]
Bladder cancer	Cell proliferation and formation of colony of cancerous cells was inhibited by Qu; Qu may be an efficient chemopreventive and chemotherapeutical agent.	[156]
Leukemia	Qu synergistically sensitizes to apoptosis numerous leukemic cell lines and B-cells isolated from patients of CLL, when associated with other proapoptotic agents.	[157]
Leukemia	It can be used as a warning factor, along with TRAIL, promoting the impact of TRAIL-induced apoptosis in KG-1 cells.	[158]
Lymphoma	Qu restores TRAIL induced cell death in resistant transformed follicular lymphoma.	[159]
Lung cancer	Antigrowth of cancer was noted in Qu treatment.	[160]
Lung cancer	Qu plays a role in enhancement of the gene expression linked with death receptor signaling and inhibition of cell cycle, but reduces the expression of genes involved in activation of NF-*κ*B.	[65]
Osteosarcoma	Qu attenuates invasion and migration of cells in MG63 and HOS cells in comparison with treatment with control medium and may have potential as a therapy for human osteosarcoma.	[161]
Osteosarcoma	It significantly reduced the mRNA expression levels of PTHR1 and consequent knockdown of PTHR1, and enhanced Qu-inhibited proliferation and invasion.	[162]
Glioblastoma	The anticancer activity of Qu was proven through repressing the PI3K-Akt pathway.	[163]
Glioblastoma	The growth of glioblastoma cells and induction of apoptosis was measured in glioblastoma cells.	[164]
Head and Neck cancer	Qu is an efficient anticancer agent in EGFR-overexpressing HNSCC.	[165]
Head and Neck cancer	The in vivo and in vitro studies have revealed that Qu has anticancer activity.	[166]
Oral cancer	The invasion and migration of human oral cancer cells is inhibited by Qu via inhibition of matrix metalloproteinase-2/-9 and NF-*κ*B signaling pathways.	[167]
Esophagus cancer	Qu-3-*O*-β-d-glucopyranoside on gastritis and reflux esophagitis in rats have been studied by Min et al. (2009), and findings demonstrate that Qu-3-*O*-β-d-3-*O*-β-d-glucopyranoside can prevent gastritis and reflux esophagitis in rats.	[168]
Melanoma	Qu quickly metabolizes tyrosinase into several compounds, which encourage anticancer activity. It suggests that Qu can be used to target melanoma.	[169]
Myeloma	Qu encourages apoptosis of myeloma cells and shows a synergetic effect with dexamethasone in vitro and in vivo xenograft models.	[105]
Retinoblastoma	The antitumor effects of Qu occur by inhibiting metastasis progression, cell proliferation, and angiogenesis, modifying cell cycle progression, encouraging apoptosis, and influencing autophagy, including retinoblastoma.	[15,170]

## Data Availability

Data is contained within the article.
